# Mass Spectrometric Identification of Ancient Proteins as Potential Molecular Biomarkers for a 2000-Year-Old Osteogenic Sarcoma

**DOI:** 10.1371/journal.pone.0087215

**Published:** 2014-01-27

**Authors:** Agnes Bona, Zoltan Papai, Gabor Maasz, Gabor A. Toth, Eva Jambor, Janos Schmidt, Csaba Toth, Csilla Farkas, Laszlo Mark

**Affiliations:** 1 Department of Analytical Biochemistry, Institute of Biochemistry and Medical Chemistry, University of Pecs, Pecs, Hungary; 2 Janos Szentagothai Research Center, University of Pecs, Pecs, Hungary; 3 Imaging Center for Life and Material Sciences, University of Pecs, Pecs, Hungary; 4 PTE-MTA Human Reproduction Research Group, Pecs, Hungary; 5 Institute of Biology, University of West Hungary, Szombathely, Hungary; 6 Department of Pathology, University Teaching Hospital Markusovszky, Szombathely, Hungary; 7 Department of Archaeology, Vas County Museums' Directorate, Szombathely, Hungary; The George Washington University, United States of America

## Abstract

Osteosarcoma is the most common primary malignant tumor of bone usually occurring in young adolescent and children. This disease has a poor prognosis, because of the metastases in the period of tumor progression, which are usually developed previous to the clinical diagnosis. In this paper, a 2000-year-old ancient bone remain with osteogenic sarcoma was analyzed searching for tumor biomarkers which are closely related to this disease. After a specific extraction SDS-PAGE gel electrophoresis followed by tryptic digestion was performed. After the digestion the samples were measured using MALDI TOF/TOF MS. Healthy bone samples from same archaeological site were used as control samples. Our results show that in the pathological skeletal remain several well known tumor biomarkers are detected such as annexin A10, BCL-2-like protein, calgizzarin, rho GTPase-activating protein 7, HSP beta-6 protein, transferrin and vimentin compared to the control samples. The identified protein biomarkers can be useful in the discovery of malignant bone lesions such as osteosarcoma in the very early stage of the disease from paleoanthropological remains.

## Introduction

Osteosarcoma is the most common primary bone tumor characterized by the production of osteoid matrix from malignant cells. It typically occurs in the long bones of the near metaphyseal growth plates of children and young adolescents [1]. The earliest known case affected a male Celt (ca. 800–600 BC) from Switzerland with a possible osteosarcoma or chondrosarcoma [2]. A possible osteosarcoma of the pelvis has been noted in a young individual from Ancient Egypt, dating to about 250 AD [3], and a well-documented case of osteosarcoma, with the typical radiographic “sunburst” pattern, has been reported in the femur of a native Peruvian dating to 800 BP [4]. Additional cases of osteosarcoma have been observed in a young female femur from the prehistoric population of Oahu in Hawaii [5], and in a zygomatic bone from the French Middle Ages [6] in a case of 17th century mandible from West Virginia [7]. Possible osteosarcomas have been detected in a young male from the Saxon necropolis of Standlake, England [8] and in medieval skulls from the Czech Republic [9] and France [10]. Probable cranial hemangiosarcoma has been documented in an elderly female from Italy, 3rd Century BC [11] and in a humerus from Peru, 12–14th Centuries AD [4] and a possible Ewing's sarcoma in a juvenile skull from Bronze Age of Tartaren, Spain [12]. Only a few cases of neoplasms have been documented in Central and South American mummies, for example a rhabdomyosarcoma (4–7th Centuries AD) in 2 children from Chile [13].

Diagnosis of cancer as well as osteogenic sarcomas from ancient human skeletal remains is not an easy task by using classic morphological methods. Therefore a biomolecular approach to diagnosis in addition to osteological examination can be beneficial [14]. Recently, proteomic profiling of human tumors has provided a better understanding of the molecular pathogenesis of neoplastic diseases and has identified novel biomarkers for early diagnosis. SELDI-TOF-MS (Surface Enhanced Laser Desorption/Ionization Time of Flight Mass Spectrometry) and protein microarray high throughput analysis enable to detect biomarkers from the serum samples of osteosarcoma patients [15]. Based on the MALDI-TOF analyses of clinical benign and osteosarcomas several biomarkers are up- or down-regulated [16]. Also MALDI-TOF analysis of human osteosarcoma MG-63 cells showed the alterations of some genes and proteins [17].

Additionally, mass spectrometry (MS) based proteomic studies of paleopathological remains have made sequence information available from subpicomolar quantities of fragmented proteins and peptides [18]–[26].

In this study, ancient proteins such as malignant bone tumor related molecular biomarkers were successfully extracted and detected from archaeological human skeletal remains by matrix-assisted laser desorption/ionization tandem time-of-flight mass spectrometry (MALDI TOF/TOF MS) for the first time. Our proteomic results can enhance the diagnosis of osteogenic tumors in ancient human skeletal remains.

## Materials and Methods

### Ethics Statement

#### Specimen numbers

Savaria, Szent Marton str. 53/grave 186 (osteosarcoma) and grave 199 (healthy control).

#### Repository information

Savaria Museum, Archaeological Collection, Kisfaludy str. 9, H-9700 Szombathely, Hungary. Department of Anthropology, University of Szeged, Kozepfasor 52, H-6726, Szeged, Hungary.

All necessary permits were obtained for the described study, which complied with all relevant regulations (National Office of Cultural Heritage permission number: 3495/2001).

### Archaeological Bone Sample

**Figure 1:**
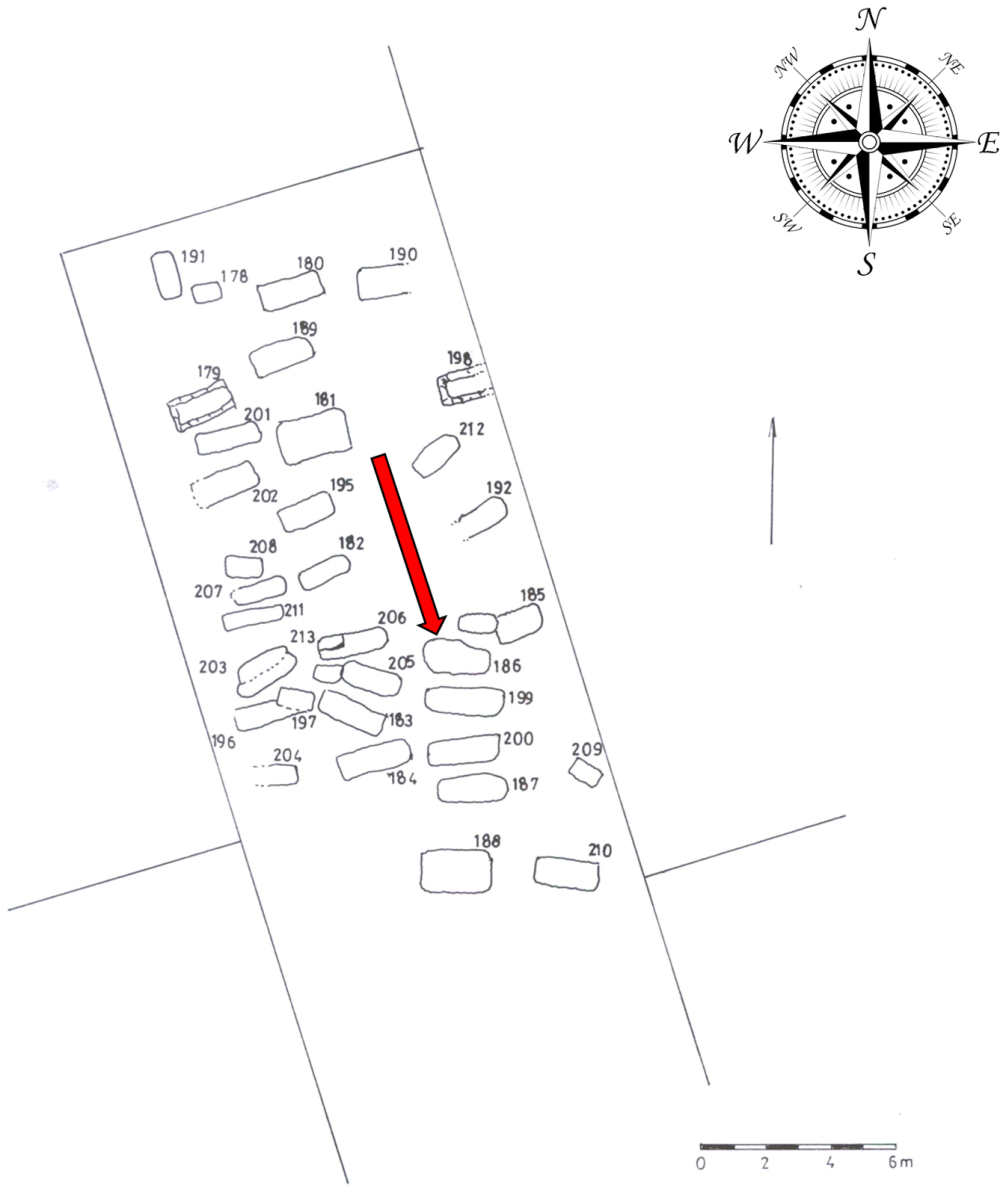
The line drawing map of the archaeological site. At this site we uncovered 33 graves from the late Roman period, arranged in four rows, very close to each other. Grave 186 (indicated with an red arrow) is located in the south-eastern section of the site, at 270–90°. The upper perimeter dimensions of the grave were 218 by 126 cm, the base 185 by 120 cm, with a depth of 48 cm.

**Figure 2:**
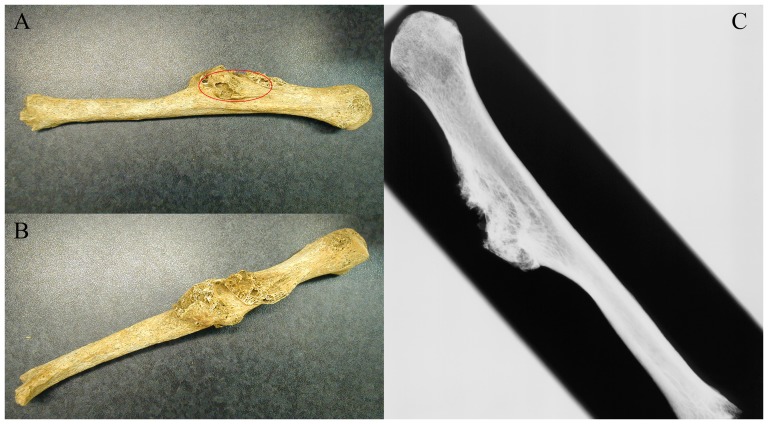
The paleoanthropological sample chosen for mass spectrometric analysis. A) and B) Anterior and posterior views of the analyzed right humerus with osteogenic sarcoma. C) X-ray radiograph of the measured human remain. The red ellipse shows the location of the sampling.

The fragmented skeleton of a 25–35-year-old female has been excavated at the Late Roman archaeological site of Szombathely (Savaria) - Szent Marton street 53, Hungary (municipal location code 6583/1) grave 186 (Figure 1). The analyzed bone sample was collected from the cortical region of the right humerus (Figure 2). The anthropological and paleopathological investigations were carried out based on Knussmann [27] and Jozsa [28]. A humerus from a non-cancerous skeletal remain from this cemetery (grave 199, adult female) was used for the investigations as a control sample. For further validation we used our previous proteomic results of some non-pathological and *M. tuberculosis* infected bone samples (Table 1 and 2).

**Table 1:** The analyzed healthy control bone samples.

Location	Grave	Age	Sex	Period
Mohacs	-	Adult	Male	Recent (forensic)
Bataszek	-	Adult	Male	Recent (forensic)
Bataszek	-	Senium	Male	Recent (forensic)
Mohacs	-	Adult	Female	Recent (forensic)
Pecs	-	Adult	Female	Recent (forensic)
Hodmezovasarhely-Gorzsa	16	Senium	Female	Neolithic
Mezokovesd Patakrajaro	4/a	Adultus	Male	Chalcolithic
Kiskundorozsma	15	Adultus	Female	Bronze Age
Algyo Barakktabor	4	Maturus	Male	Scythian
Szegvar Oromdulo	918	Adultus	Female	Sarmatian
Kishomok	89	Maturus	Female	Gepids
Szegvar Oromdulo	740	Adultus	Female	Early Avar
Szekkutas Kapolnadulo	30	Adultus	Female	Late Avar
Kiskundorozsma	100	Senium	Male	Hungarian Conquest
Esztergalyhorvath	284	Adultus	Female	Hungarian Conquest
Kecskemet Torokfaj	65	Maturus	Female	Arpad Age, X-XI^th^ AD
Szegvar Oromdulo	617	Maturus	Female	Arpad Age, XI^th^ AD
Derekegyhaza Ibolyas	13	Senium	Male	XI-XII^th^ AD
Csengele Bogarhat	57	Adultus	Male	XIII^th^ AD
Kecskemet Ferences church	350	Adultus	Female	XIV-XV^th^ AD

**Table 2:** The measured pathological control (*Mycobacterium tuberculosis* infected) archaeological bone samples.

Location	Grave	Age	Sex	Period
Sukosd-Sagod	19	Adult	Female	VII-VIII^th^ AD
Sukosd-Sagod	19	Adult	Female	VII-VIII^th^ AD
Bacsalmas-Homokbanya	39	Maturus	Male	XVII^th^ AD
Bacsalmas-Homokbanya	39	Maturus	Male	XVII^th^ AD
Belmegyer-Csomoki hill	65	Maturus	Female	VIII^th^ AD
Belmegyer-Csomoki hill	65	Maturus	Female	VIII^th^ AD
Csongrad-Elles	183	Maturus	Male	XI-XIII^th^ AD
Csongrad-Elles	183	Maturus	Male	XI-XIII^th^ AD
Csongrad-Elles	183	Maturus	Male	XI-XIII^th^ AD
Csongrad-Elles	183	Maturus	Male	XI-XIII^th^ AD
Csongrad-Felgyo	205	Adult	Female	VIII^th^ AD
Csongrad-Felgyo	205	Adult	Female	VIII^th^ AD

### Extraction of Ancient Proteins

The sample preparation, separation and mass spectrometric analysis is of a vital importance for the quality of the results. In our previous study we developed an optimized workflow for proteomic analysis of ancient proteins [19]. Here, we used this method with some modifications. Briefly, the bone fragments were washed to remove contaminants with phosphate buffer saline (PBS) and distillated water. Bone powder was ground by hand with an agate mortar, the particle size was ∼0.2 mm. Next, 100 mg of crude bone powder was decalcified with 1.00 ml of 0.5 M EDTA (pH = 8.0), the pellet was resuspended with 100 µl of 6 M guanidine-HCl in 0.1 M Tris (pH = 7.5) at room temperature. The extraction of the proteins was carried out by continuous shaking at 4°C for 8 hours with the presence of protease inhibitor cocktail (Sigma Aldrich Kft., Budapest, Hungary). The protein extract was purified by using C_18_ solid phase extraction (SPE) cartridge. For this purification step a homemade octadecylsilane modified silica-based stationary phase was used with average particle size of 5 µm and pore size of 120 Å. The stationary phase was activated with an aqueous 0.1% TFA solution, the loaded protein extract was washed with 100 µl of 2% acetonitrile in 0.1% TFA three times, then the proteins were eluted by 50 µl of 50% acetonitrile in 0.1% TFA solution. The solution was lyophilized to powder and stored at −86°C until further processing.

The bone sample with tumorous lesion was measured in eight technical replicates.

### SDS-PAGE Gel Electrophoresis and Enzymatic Digestion

100 µL of protein extract of the archaeological sample in 20 mM Tris/HCl buffer, pH 7.4 containing 3 mM EDTA, 5 mM betamercaptoethanol and 1% sodium dodecylsulfate (SDS) was homogenized by using Ultra Turrax homogenizer. After the addition of 1% bromphenolblue, the samples were boiled for 2 minutes and clarified by centrifuging (8000 g for 2 min). Sodium dodecylsulfate-polyacrylamide gel electrophoresis (SDS-PAGE) was carried out on 12% gel by Laemmli's method. A low molecular weight calibration kit (Pharmacia) was used for estimation of the molecular weight. To increase the quality of the separation and visibility of the spots the gel was run at 4°C. Gels were stained with Coomassie brillant blue R-250 and destained with a solution containing 5% (v/v) acetic acid and 16% (v/v) methanol.

The spots of the overexpressed proteins (compared to the healthy archaeological samples) were excised from the gel with a razorblade, placed in Eppendorf tubes, and destained by washing three times for 10 min in 200 µL of 50% (v/v) acetonitrile solution containing 50 mM NH_4_HCO_3_. Proteins were then reduced by 50 µL of 20 mM dithiotreitol, 100 mM NH_4_HCO_3_ and acetonitrile 5% for 1 h at 55°C and alkylated in 50 µL of 20 mM iodoacetamide solution. The gel pieces were dehydrated at room temperature by a Speed Vac Concentrator (Speed Vac Plus, SC100A, Savant) and covered with 10 µL of modified trypsin (Promega, Madison, WI, sequencing grade) (0.04 mg×mL^−1^) in Tris buffer (2.5 mM, pH 8.5) and left at 37°C overnight. The excised spots were crushed and peptides were extracted in an ultrasonic bath (15 min) with 15 µL aqueous solution of acetonitrile and formic acid (49/50/1 v/v/v).

### MALDI TOF/TOF Mass Spectrometry-based Identification of the Ancient Proteins

After extraction the peptide solutions were lyophilized and redissolved in 0.1% trifluoroacetic acid (TFA). The aqueous solutions of the lyophilized protein digests were concentrated and desalted by using C_18_ ZipTip SPE pipette tips (Millipore Kft, Budapest, Hungary) then the purified peptides were eluted directly onto the target plate (MTP 384 massive target T, Bruker Daltonics, Bremen, Germany) by using of 3 µL of a saturated matrix solution, prepared fresh every day by dissolving α-cyano-4-hydroxycinnamic acid (CHCA) in acetonitrile/0.1% TFA (1/2, v/v). The mass spectrometer used in this work was an Autoflex II TOF/TOF (Bruker Daltonics, Bremen, Germany) operated in reflectron mode for peptide mass fingerprinting (PMF) or LIFT mode for LID (laser induced decay) and CID (collision induced decay). The FlexControl 2.4 software was used to control the instrument. The accelerating voltage was set to 20.00 kV. The instrument uses a 337 nm nitrogen laser (model MNL-205MC, Lasertechnik Berlin GmbH., Berlin, Germany). External calibration was performed in each case using Bruker Peptide Calibration Standard (#206195 Peptide Calibration Standard, Bruker Daltonics, Bremen, Germany). Peptide masses were acquired in the range of m/z 700 to m/z 5000. Each spectrum was produced by accumulating data from 1000 consecutive laser shots. Singly charged monoisotopic peptide masses were searched against Swiss-Prot and NCBI nr databases (last accessed: 11/19/2012) by utilizing the MASCOT database search engine (version 2.2) (www.matrixscience.com, Matrix Science Ltd., London, UK) and Bruker ProteinScape server 2.1 (Bruker Daltonics, Bremen, Germany). Maximum one missed tryptic cleavage was considered, and the mass tolerance for monoisotopic peptide masses was set to 80 ppm. Carbamidomethylation was set as global modification while methionine oxidation was set as variable modification. Additionally LID and CID fragmentation of the matched peptides were carried out for MALDI TOF/TOF to provide further evidence for the presence of the identified proteins.

### Statistical Analysis

To demonstrate the predictive value of the identified biomarkers the mass spectrometric results were statistically evaluated by ClinProTools 2.2 (Bruker Daltonics, Bremen, Germany) clustering software. Multiple spectra of the analyzed bone samples from different sample cohorts, such as osteosarcoma, non-pathological and pathological (tuberculotic) control samples were distinguished together. Recalibration, spectral alignment, peak normalization, peak detection and peak area calculation of spectra were carried out automatically by ClinProTools. A logistic regression model was performed to identify the significant predictive peaks on the basis of the normalized peak areas. Wilcoxon signed-rank test was used for non-parametric statistical analysis of the different sample cohorts.

## Results and Discussion

**Figure 3:**
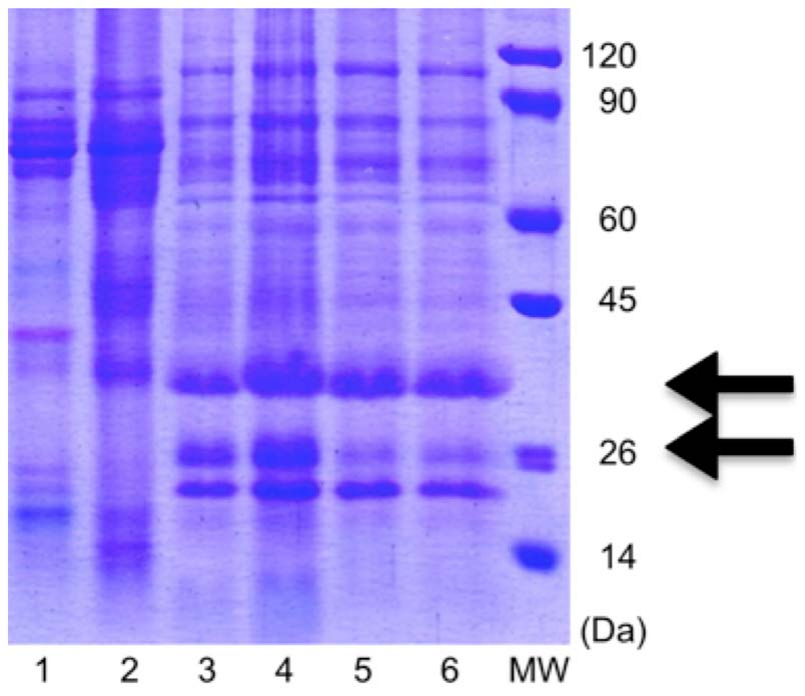
Characteristic 1D SDS PAGE electrophoretogram of healthy control and the pathological bone samples. Lanes 1 and 2 are healthy control samples from Hodmezovasarhely-Gorzsa and Szegvar-Oromdulo, lanes 3–6 are osteosarcoma samples. Arrows show the spots of Annexin A10 (37.3 kDa) and Vimentin (26.8 kDa). The parameters of the separation are mentioned in the text.

This work focused on the identification of possible protein biomarkers of osteogenic sarcoma from a 2000-year-old anthropological sample. The proteins were separated by 1D gel electrophoresis and the interested spots were enzymatically digested (Figure 3). The tryptic peptides were analyzed by MALDI TOF/TOF MS and the identification of the resulted proteins was carried out using a PMF or MS/MS search. Based on our results several known, previously published osteosarcoma or tumor related proteins and gene products were detected from the ancient pathological bone sample (Table 3).

**Table 3:** The identified up-regulated proteins from 2000-year-old osteogenic sarcoma.

Accession	Name	MW [kDa]	Peptides	SC [%]
AK1A1_HUMAN	Alcohol dehydrogenase (NADP+)	36,5	5	14,2
gi|225939	aldehyde reductase	36,3	5	14,2
gi|48762937	annexin A10	37,3	8	27,2
gi|62087532	arginine/serine-rich splicing factor 6 variant	31,8	6	27,6
ARI5B_HUMAN	AT-rich interactive domain-containing protein 5B	132,2	11	13,0
gi|33878074	BAT2 protein	17,1	5	29,9
gi|49456879	BCL2A1	20,3	7	44,0
VMDL3_HUMAN	Bestrophin-4	76,1	7	14,5
gi|882391	bone morphogenic protein type II receptor	59,9	6	13,2
S10AB_HUMAN	Calgizzarin (S100 calcium-binding protein A11)	11,7	4	43,8
CAN7_HUMAN	Calpain-7	92,6	7	12,3
K1C10_HUMAN	Cytokeratin 10	59,5	14	23,1
gi|33188433	deleted in liver cancer 1 isoform 1	170,5	13	7,9
gi|28704113	DHX8 protein	138,7	13	13,8
G59435	DLC-1	122,7	10	10,9
DNL3_HUMAN	DNA ligase III	102,6	10	16,3
TDT_HUMAN	DNA nucleotidylexotransferase	58,4	9	22,2
MP2K6_HUMAN	Dual specificity mitogen-activated protein kinase kinase 6	37,5	7	23,4
DTNA_HUMAN	Dystrobrevin alpha	83,9	6	10,5
gi|15010856	galectin-12 isoform d	30,0	5	30,5
GCC2_HUMAN	GRIP and coiled-coil domain-containing protein 2	184,5	18	12,3
gi|40555827	heat shock factor protein 2 isoform c	27,0	7	23,9
HSPB6_HUMAN	Heat-shock protein beta-6	17,1	5	35,0
CAC10772	Immunoglobulin heavy chain variable region	12,4	5	65,5
gi|17318569	keratin 1	66,0	15	25,8
Q8N175_HUMAN	Keratin 10	58,8	14	23,5
gi|31559819	keratin 25C	49,8	8	17,2
gi|47132620	keratin 2a	65,4	9	16,4
CAA82315	keratin 9	62,1	11	16,9
A44861	keratin, 67K type II epidermal	65,8	9	16,3
K1C9_HUMAN	Keratin, type I cytoskeletal 9	61,9	11	20,7
AAP97338	Methyl-CpG-binding domain protein 4	60,9	13	16,3
NEBL_HUMAN	Nebulette	116,4	14	16,7
NRAP_HUMAN	Nebulin-related-anchoring protein	197,0	24	14,9
gi|4506335	parvalbumin	12,1	8	56,4
PRVA_HUMAN	Parvalbumin alpha	11,9	8	56,9
gi|39653323	PHD finger protein 20-like 1 isoform 1	47,7	7	15,8
gi|39653321	PHD finger protein 20-like 1 isoform 3	16,6	6	42,0
gi|31873386	phospholipase C	39,7	7	30,7
gi|346323	phosphoprotein phosphatase (EC 3.1.3.16) X catalytic chain	35,1	6	26,1
PDIP2_HUMAN	Polymerase delta-interacting protein 2	42,0	7	22,0
CAF00150	Proteasome subunit beta type 3	16,6	5	34,9
gi|565647	proteasome subunit HsC10-II	22,9	5	25,4
PARK7_HUMAN	Protein DJ-1 (Oncogene DJ1)	19,9	5	32,3
gi|55859594	PTAR1 protein	32,4	7	30,1
gi|4960030	Rab GDP dissociation inhibitor beta	41,0	6	22,0
gi|39841018	RAB GTPase activating protein 1-like	92,5	8	10,3
gi|537327	receptor tyrosine kinase	18,5	6	30,0
gi|2665850	rheumatoid factor RF-ET7	10,9	6	74,5
RHG07_HUMAN	Rho GTPase-activating protein 7	122,7	10	10,9
gi|41152086	serine (or cysteine) proteinase inhibitor, clade B, member 6	42,6	9	29,3
gi|38382764	SET-binding protein isoform b	26,4	7	25,6
gi|46250431	Transcription factor NRF	77,7	11	20,4
gi|62088924	Transducin-like enhancer of split 3 splice variant 1 variant	22,9	6	37,1
gi|37747855	Transferrin	77,0	10	21,9
gi|4507659	translocated promoter region (to activated MET oncogene)	265,4	24	12,0
gi|4827050	ubiquitin specific protease 14	56,0	8	20,6
gi|71774197	ubiquitin specific protease 47	147,1	10	13,4
gi|34532272	unnamed protein product (homolog of CCDC144A protein)	56,3	12	21,9
gi|21754902	unnamed protein product (homolog of Zinc finger protein 781)	17,8	7	48,7
gi|57471648	vimentin	26,8	8	38,2
WBP4_HUMAN	WW domain-binding protein 4	42,5	7	15,7
ZN224_HUMAN	Zinc finger protein 224	82,2	10	19,4
gi|74355161	Zinc finger protein 624	85,6	11	22,5

(Mascot score>50).

The identified annexins (ANXs) are calcium and phospholipid binding proteins, they play a crucial role in the exocytic and endocytic transport, regulation of cell growth, proliferation and apoptosis. It is well known, that the increased level of ANX (ANXA-10) indicates tumor progression [29], [30]. The B-cell lymphoma 2-related protein A1 (BCL2A1) is a member of BCL2 proteins. BCL2A1 is responsible for the separation of pro-apoptotic BCL2 proteins. BCL2A1 shows an elevated level in case of different cancer types such as leukemia and lymphoma, also connected with autoimmunity and therapy resistance of different tumors [31]–[34]. Calgizzarin (S100A11) belongs to the calcium binding proteins S100 family, involved in cell growth, motility and differentiation. Calgizzarin has been correlated with tumor progression and metastasis [35], [36]. DLC1 is a known tumor suppressor, acting through Rho GTPase-activating protein (RhoGAP), which is involved in the proliferation and migration of tumor cells, induces apoptosis in vitro [37]–[39]. Heat-shock protein's (HSP's) expression increases in case of thermal, physiological or other stress factors allowing the cells to survive lethal conditions. HSP's play key role in the apoptotic and cell death process (inhibition of caspase activation). Elevated level of HSP beta-6 was found in clinical samples of patients who suffered from osteosarcoma [40], [41]. DJ-1 protein is a mitogen-dependent oncogene involved in ras-related signal transduction pathway. Overexpression of DJ-1 indicates tumorous mutation [42], [43]. The RhoGAP family proteins play an important role in regulating cell migration, cell morphology and cytoskeletal organization. Down regulation of RhoGAP proteins decrease the tumor suppressive effect [44]. Transferrin is a member of iron-binding blood plasma glycoproteins, that is responsible for the regulation of the free iron content in the blood. Elevated level of transferrin is correlated with tumorous diseases such as osteosarcoma [40], [45]. The expression of a cytoskeletal intermediate filament protein vimentin (VIM) was also shown to increase in case of osteosarcoma [40], [45]–[47]. VIM is considered to be a tumor biomarker, as it is promoting the metastatic spread of the tumor cells. In this study, some keratins were identified as well. The origin and the importance of these proteins are not well known, probably the identified keratins are from recent or contemporary contaminations. However, the up-regulation of cytokeratins has been published in U2OS osteosarcoma specific cell line [43].

**Figure 4:**
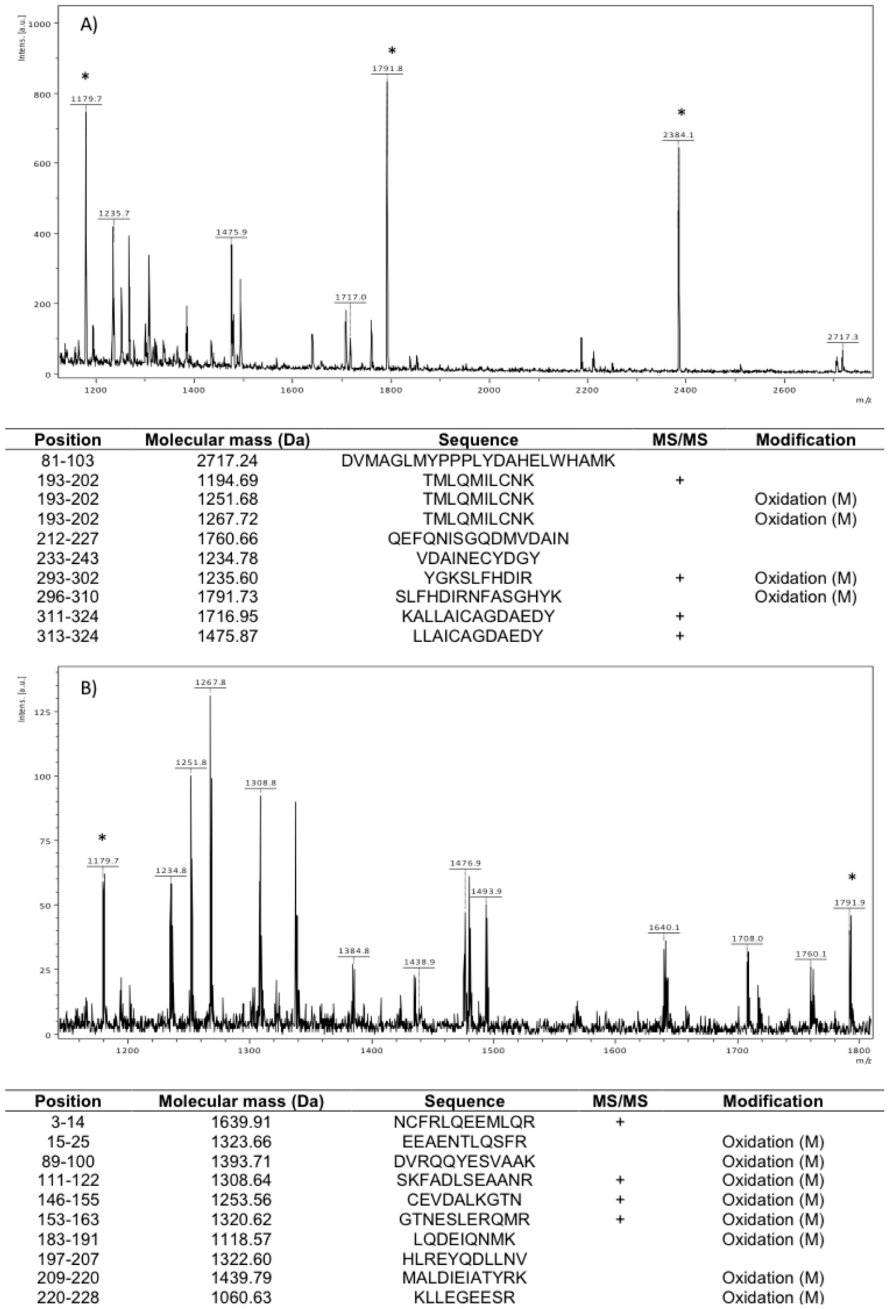
Representative mass spectra and the list of the identified tryptic peptides of two identified tumor biomarkers. A) Annexin A10, B) Vimentin. Some keratin contamination has been detected in the sample, the tryptic peptides of keratin were used as internal calibration standards and the peaks are marked with asterisk.

Representative mass spectra and the list of the identified tryptic peptides of annexin A10 and vimentin are showed on Figure 4. The results show characteristic peaks of the identified biomarkers, however some keratin peaks are present on the spectra. This pollution is common from ancient bone tissue, but various types of keratins are up-regulated in different tumors as well.

**Figure 5:**
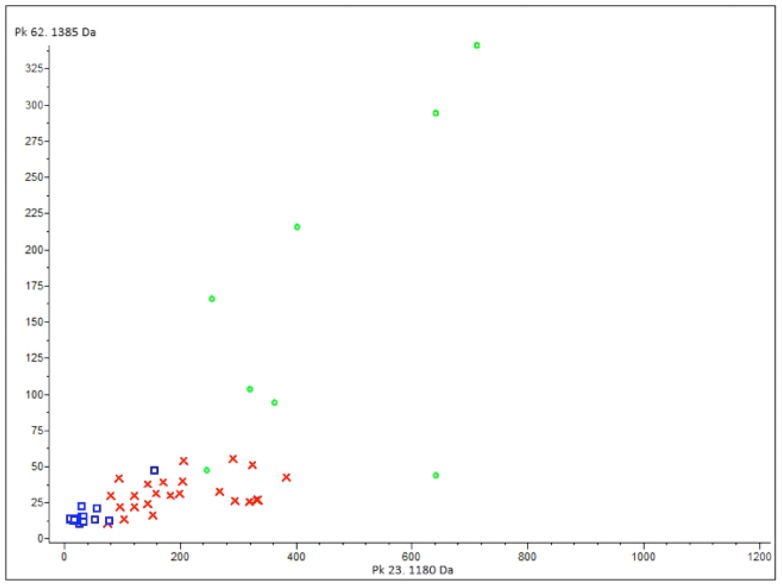
ClinProTools-based Wilcoxon non-parametric statistical test of different sample cohorts. Cluster analysis from sample sets of the osteosarcoma (green), healthy control sample (red) and tuberculotic control sample (blue) groups using the peptide peaks with m/z 1180 and 1385. The x-and y-axes correspond to the relative intensities of the peptide peaks.

Based on our statistical analysis the different sample cohorts such as osteosarcoma, healthy control and tuberculotic control could be distinguished, therefore the spectral profile of the samples with osteosarcoma is significantly different as the other two group's profiles (Figure 5). The predictive peaks are showed in Table S1, where the statistical parameters are given for the most predictive molecular masses.

## Conclusions

The aim of our study was to find potential tumor biomarkers for ancient osteosarcoma using healthy bone samples as a control. Certain proteins, which are characteristic for tumorous disease can be extracted from the diseased cells, were presumably transported earlier by the blood and absorbed to the bone hydroxyapatite [48]. Using an appropriate extraction method, developed by our research group, followed by SDS PAGE, tryptic digestion and MALDI TOF/TOF, we were able to identify several proteins that were tightly connected to tumorous mutations. The overexpression of annexin A10 protein, BCL-2-like protein, calgizzarin, HSP beta-6, RhoGAP-activating protein 7, transferrin, and vimentin among others referred to healthy samples may indicate the presence of tumor in bones. In this study, we demonstrated that the peptide profile of the samples with osteosarcoma is statistically unique and it could be distinguished from other sample cohorts.

On the basis of our results the proteomic analyses could indicate the presence of osteosarcoma in bone tissues. Our findings showed that the well known, osteosarcoma-related clinical protein biomarkers are detectable in the investigated 2000-year-old tumorous skeletal remain. In the future, additional comparative proteomic investigations are needed for further early stage biomarker discovery of ancient primary bone cancer.

## Supporting Information

Table S1 The statistical parameters of the mass spectrometric results.
(DOCX)
