# Correction: Mass Spectrometric Identification of Ancient Proteins as Potential Molecular Biomarkers for a 2000-Year-Old Osteogenic Sarcoma

**DOI:** 10.1371/journal.pone.0103862

**Published:** 2014-07-24

**Authors:** The PLOS ONE Staff

There are several errors in Figure 4. The authors have provided a corrected version of Figure 4 below.

**Figure 4:**
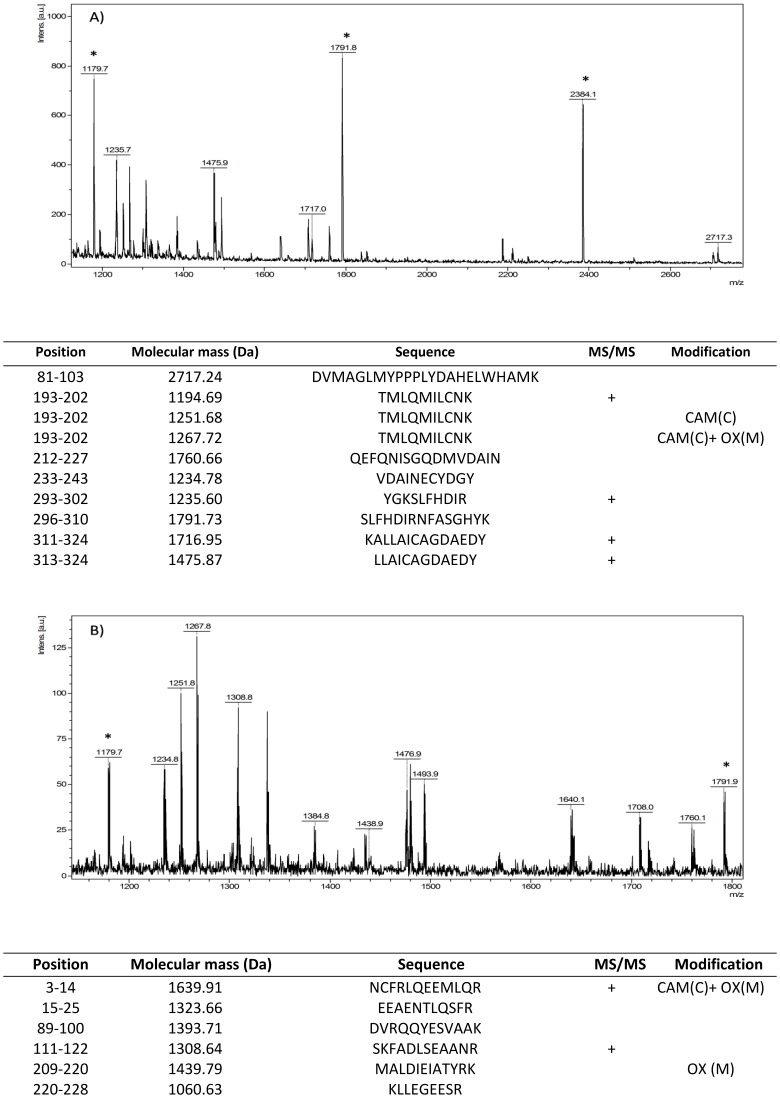
Representative mass spectra and the list of the identified tryptic peptides of two identified tumor biomarkers. A) Annexin A10, B) Vimentin. Some keratin contamination has been detected in the sample, the tryptic peptides of keratin were used as internal calibration standards and the peaks are marked with asterisk. The used abbreviations: CAM - carbamidomethylation of cystein , OX - oxidation of methionine.
