# Supplementary material for: Mass Spectrometric Identification of Ancient Proteins as Potential Molecular Biomarkers for a 2000-Year-Old Osteogenic Sarcoma
Source: PLoS One. 2014 Jan 27;9(1):e87215. doi: 10.1371/journal.pone.0087215 (PMC3903643; doi:10.1371/journal.pone.0087215)
Supplement: Table S1 — The statistical parameters of the mass spectrometric results. (DOCX) [file pone.0087215.s001.docx]

**Supporting Information Table 1: The statistical parameters of the mass spectrometric results.**

Detailed statistical results of predictive mass spectrometric peaks. The statistical analysis made by ClinProTools software (Bruker Daltonics, Bremen, Germany).

DAve – Difference between the maximum and minimum average peak area of all classes, PTTA – P-value of T-test or ANOVA, PWKW – P-value of Wilcoxon or Kruskal-Walis test, PAD – P-value of Anderson-Darling test.

| **Mass [Da]** | **DAve** | **PTTA** | **PWKW** | **PAD** |
| --- | --- | --- | --- | --- |
| 1179.76 | 96.73 | 0.00066 | 0.00000794 | < 0.000001 |
| 1384.80 | 34.64 | 0.00159 | 0.00000794 | < 0.000001 |
| 1717.86 | 19.76 | 0.00106 | 0.00000794 | < 0.000001 |
| 1488.74 | 8.31 | 0.00148 | 0.00000885 | < 0.000001 |
| 1194.61 | 23.98 | 0.00281 | 0.00000948 | < 0.000001 |
| 1476.88 | 50.4 | 0.00116 | 0.0000158 | < 0.000001 |
| 1277.86 | 29.86 | 0.0204 | 0.0000158 | < 0.000001 |
| 1839.11 | 11.10 | 0.00066 | 0.0000158 | 0.00000849 |
| 1490.72 | 5.05 | 0.00066 | 0.0000158 | 0.0000151 |
| 1263.80 | 7.51 | 0.0023 | 0.0000183 | < 0.000001 |
| 1308.71 | 95.91 | 0.00829 | 0.0000195 | < 0.000001 |
| 1476.01 | 53.79 | 0.00066 | 0.0000225 | < 0.000001 |
| 1165.68 | 14.51 | 0.000962 | 0.000034 | 0.0000076 |
| 1700.84 | 4.83 | 0.00159 | 0.0000433 | < 0.000001 |
| 1323.7 | 11.75 | 0.0166 | 0.0000438 | < 0.000001 |
| 1701.93 | 1.99 | 0.00066 | 0.0000504 | 0.000958 |
| 1487.98 | 10.85 | 0.00529 | 0.0000538 | < 0.000001 |
| 1264.72 | 7.06 | 0.00529 | 0.0000723 | < 0.000001 |
| 1379.83 | 3.43 | 0.00333 | 0.0000723 | < 0.000001 |
| 1365.83 | 14.44 | 0.00219 | 0.0000746 | < 0.000001 |
| 1874.06 | 4.28 | 0.0162 | 0.0000795 | < 0.000001 |
| 1265.63 | 5.16 | 0.00529 | 0.000111 | 0.00000207 |
| 1235.65 | 63.01 | 0.00225 | 0.000129 | < 0.000001 |
| 1833.87 | 6.48 | 0.00829 | 0.00013 | < 0.000001 |
| 1702.94 | 6.42 | 0.00829 | 0.000149 | < 0.000001 |
| 1366.60 | 12.90 | 0.00955 | 0.000158 | < 0.000001 |
| 1391.72 | 5.89 | 0.00159 | 0.000158 | < 0.000001 |
| 1381.69 | 4.60 | 0.00826 | 0.000158 | < 0.000001 |
| 1380.72 | 4.91 | 0.0227 | 0.000189 | < 0.000001 |
| 1995.10 | 10.21 | 0.0204 | 0.000221 | < 0.000001 |
| 1300.74 | 20.88 | 0.00829 | 0.000234 | < 0.000001 |
| 1201.79 | 15.07 | 0.0339 | 0.000292 | < 0.000001 |
| 1316.75 | 5.32 | 0.00525 | 0.000292 | < 0.000001 |
| 1605.89 | 11.12 | 0.00829 | 0.000318 | < 0.000001 |
| 1849.00 | 4.51 | 0.0123 | 0.000324 | < 0.000001 |
| 2691.35 | 3.13 | 0.0207 | 0.000358 | < 0.000001 |
| 1033.6 | 6.28 | 0.0104 | 0.00038 | < 0.000001 |
| 1465.83 | 42.24 | 0.0176 | 0.000439 | < 0.000001 |
| 1340.62 | 11.13 | 0.0123 | 0.000439 | < 0.000001 |
| 1305.79 | 6.81 | 0.0213 | 0.000439 | < 0.000001 |
| 1506.83 | 2.24 | 0.00245 | 0.000439 | < 0.000001 |
| 1302.61 | 10.99 | 0.00973 | 0.000444 | < 0.000001 |
| 1392.67 | 3.57 | 0.00416 | 0.000465 | < 0.000001 |
| 2961.81 | 12.87 | 0.0428 | 0.000469 | < 0.000001 |
| 1131.65 | 6.34 | 0.0648 | 0.000473 | < 0.000001 |
| 1568.76 | 10.53 | 0.0283 | 0.000581 | < 0.000001 |
| 1360.73 | 3.16 | 0.0208 | 0.000581 | < 0.000001 |
| 1358.71 | 9.12 | 0.0104 | 0.000703 | < 0.000001 |
| 1330.66 | 4.76 | 0.0382 | 0.000882 | < 0.000001 |
| 1439.90 | 8.54 | 0.0204 | 0.000918 | < 0.000001 |
| 1333.61 | 3.64 | 0.0183 | 0.00112 | < 0.000001 |
| 1350.71 | 6.73 | 0.0386 | 0.00115 | < 0.000001 |
| 2369.3 | 1.65 | 0.00281 | 0.00122 | < 0.000001 |
| 1562.97 | 19.09 | 0.0115 | 0.00128 | < 0.000001 |
| 1332.76 | 3.65 | 0.0166 | 0.00131 | < 0.000001 |
| 1074.58 | 6.19 | 0.0278 | 0.00132 | < 0.000001 |
| 1548.72 | 13.66 | 0.0292 | 0.00135 | < 0.000001 |
| 1792.67 | 62.30 | 0.00444 | 0.00136 | < 0.000001 |
| 1657.92 | 5.57 | 0.0162 | 0.00136 | < 0.000001 |
| 1349.84 | 5.79 | 0.03 | 0.00137 | < 0.000001 |
| 1344.73 | 2.53 | 0.00914 | 0.00148 | < 0.000001 |
| 1291.76 | 3.82 | 0.104 | 0.00153 | < 0.000001 |
| 3067.40 | 3.37 | 0.062 | 0.00155 | < 0.000001 |
| 1621.83 | 4.98 | 0.00829 | 0.0016 | < 0.000001 |
| 1852.93 | 10.55 | 0.00416 | 0.00162 | < 0.000001 |
| 1163.64 | 7.26 | 0.0188 | 0.00162 | < 0.000001 |
| 1407.73 | 6.35 | 0.0208 | 0.00162 | < 0.000001 |
| 1393.76 | 6.34 | 0.0191 | 0.00174 | < 0.000001 |
| 1425.93 | 4.59 | 0.023 | 0.00203 | < 0.000001 |
| 1037.51 | 11.48 | 0.0204 | 0.00215 | < 0.000001 |
| 1923.83 | 4.10 | 0.00066 | 0.00215 | < 0.000001 |
| 3118.84 | 3.78 | 0.0701 | 0.00215 | < 0.000001 |
| 1337.84 | 74.39 | 0.0179 | 0.00217 | < 0.000001 |
| 2511.34 | 2.22 | 0.00262 | 0.00228 | 0.000424 |
| 1434.97 | 21.93 | 0.0188 | 0.00237 | < 0.000001 |
| 1157.76 | 5.94 | 0.0448 | 0.00241 | < 0.000001 |
| 1655.98 | 5.48 | 0.0467 | 0.00246 | < 0.000001 |
| 2569.46 | 1.40 | 0.0188 | 0.00251 | < 0.000001 |
| 2330.39 | 1.58 | 0.00829 | 0.00252 | < 0.000001 |
| 1267.89 | 185.07 | 0.0378 | 0.00252 | < 0.000001 |
| 2706.54 | 22.59 | 0.0117 | 0.00252 | < 0.000001 |
| 1547.82 | 21.48 | 0.0551 | 0.00252 | < 0.000001 |
| 1435.69 | 16.80 | 0.0353 | 0.00252 | < 0.000001 |
| 1400.86 | 6.81 | 0.0493 | 0.00252 | < 0.000001 |
| 1249.76 | 5.12 | 0.0204 | 0.00271 | < 0.000001 |
| 1090.64 | 6.28 | 0.00829 | 0.00273 | 0.0000211 |
| 1808.93 | 1.98 | 0.0852 | 0.00273 | < 0.000001 |
| 1741.96 | 4.10 | 0.0523 | 0.00284 | < 0.000001 |
| 1478.74 | 56.38 | 0.00281 | 0.0029 | < 0.000001 |
| 2005.08 | 14.47 | 0.0104 | 0.00321 | < 0.000001 |
| 1581.78 | 15.93 | 0.00632 | 0.00334 | < 0.000001 |
| 1523.92 | 3.06 | 0.0448 | 0.00356 | < 0.000001 |
| 1507.81 | 2.60 | 0.0148 | 0.00412 | < 0.000001 |
| 2116.19 | 16.56 | 0.00303 | 0.00428 | < 0.000001 |
| 1891.12 | 1.12 | 0.104 | 0.00449 | < 0.000001 |
| 1013.61 | 6.47 | 0.0482 | 0.00482 | < 0.000001 |
| 1320.79 | 13.43 | 0.0552 | 0.005 | < 0.000001 |
| 1592.01 | 4.30 | 0.0452 | 0.005 | < 0.000001 |
| 1251.91 | 83.66 | 0.0628 | 0.00514 | < 0.000001 |
| 1664.87 | 2.03 | 0.0208 | 0.00541 | < 0.000001 |
| 2456.35 | 4.33 | 0.0467 | 0.00562 | < 0.000001 |
| 1141.57 | 3.50 | 0.0448 | 0.00635 | 0.0000678 |
| 1060.28 | 13.32 | 0.0188 | 0.00638 | < 0.000001 |
| 2317.26 | 3.25 | 0.0135 | 0.00649 | < 0.000001 |
| 2058.13 | 12.58 | 0.00266 | 0.00697 | < 0.000001 |
| 1479.84 | 38.98 | 0.0428 | 0.00711 | < 0.000001 |
| 1440.71 | 5.78 | 0.051 | 0.00755 | < 0.000001 |
| 1119.48 | 15.72 | 0.0248 | 0.00837 | < 0.000001 |
| 2217.10 | 11.12 | 0.0162 | 0.00914 | < 0.000001 |
| 1963.00 | 25.34 | 0.00219 | 0.00929 | < 0.000001 |
| 1780.90 | 2.27 | 0.14 | 0.00929 | < 0.000001 |
| 2728.60 | 2.87 | 0.0105 | 0.0109 | 0.00000678 |
| 3102.98 | 1.99 | 0.0628 | 0.0109 | < 0.000001 |
| 1777.10 | 4.54 | 0.104 | 0.0109 | < 0.000001 |
| 1455.80 | 1.88 | 0.0261 | 0.0112 | < 0.000001 |
| 1095.65 | 11.72 | 0.0964 | 0.0112 | < 0.000001 |
| 1477.80 | 85.01 | 0.00525 | 0.0113 | < 0.000001 |
| 1584.87 | 3.88 | 0.0188 | 0.0113 | < 0.000001 |
| 1317.61 | 3.46 | 0.0391 | 0.0113 | < 0.000001 |
| 1761.00 | 19.02 | 0.0283 | 0.0124 | < 0.000001 |
| 2187.18 | 6.35 | 0.0651 | 0.0132 | < 0.000001 |
| 1003.59 | 21.43 | 0.0829 | 0.0141 | < 0.000001 |
| 1459.90 | 11.70 | 0.0448 | 0.0141 | < 0.000001 |
| 1634.03 | 3.91 | 0.0951 | 0.0148 | < 0.000001 |
| 1460.67 | 7.17 | 0.0852 | 0.0163 | < 0.000001 |
| 2498.32 | 1.62 | 0.0462 | 0.0174 | < 0.000001 |
| 2152.10 | 1.46 | 0.135 | 0.0177 | < 0.000001 |
| 1586.84 | 8.58 | 0.0944 | 0.0181 | < 0.000001 |
| 1501.86 | 2.58 | 0.0628 | 0.0183 | < 0.000001 |
| 1585.96 | 3.21 | 0.0248 | 0.0187 | < 0.000001 |
| 1422.71 | 3.90 | 0.0852 | 0.0199 | < 0.000001 |
| 1352.70 | 3.05 | 0.0551 | 0.0203 | < 0.000001 |
| 1491.77 | 2.51 | 0.0455 | 0.0224 | 0.0000661 |
| 2106.99 | 1.53 | 0.0261 | 0.0225 | 0.0000653 |
| 2073.17 | 1.81 | 0.502 | 0.0246 | 0.0000876 |
| 1082.28 | 8.21 | 0.0797 | 0.0248 | 0.0000154 |
| 3185.88 | 1.51 | 0.0729 | 0.0256 | < 0.000001 |
| 1708.78 | 19.51 | 0.127 | 0.0284 | < 0.000001 |
| 1066.20 | 10.01 | 0.115 | 0.0346 | 0.002 |
| 2236.29 | 8.93 | 0.0628 | 0.039 | < 0.000001 |
| 1126.48 | 4.07 | 0.0729 | 0.0558 | < 0.000001 |
| 2472.31 | 1.24 | 0.0868 | 0.0577 | < 0.000001 |
| 1105.85 | 26.11 | 0.0261 | 0.0661 | < 0.000001 |
| 1509.83 | 1.39 | 0.0424 | 0.0668 | 0.000256 |
| 2385.10 | 34.07 | 0.0213 | 0.0752 | < 0.000001 |
| 2718.93 | 10.22 | 0.0669 | 0.0816 | < 0.000001 |
| 2911.43 | 5.22 | 0.104 | 0.0816 | < 0.000001 |
| 1137.60 | 4.43 | 0.141 | 0.0816 | < 0.000001 |
| 1098.63 | 12.23 | 0.131 | 0.0935 | < 0.000001 |
| 1406.86 | 3.36 | 0.104 | 0.0966 | < 0.000001 |
| 2400.16 | 3.20 | 0.0467 | 0.103 | < 0.000001 |
| 1493.97 | 35.94 | 0.14 | 0.108 | 0.00000275 |
| 1639.96 | 23.62 | 0.104 | 0.109 | < 0.000001 |
| 1045.67 | 1.99 | 0.421 | 0.112 | 0.00000922 |
| 2871.24 | 3.94 | 0.051 | 0.136 | < 0.000001 |
| 1388.74 | 3.00 | 0.0828 | 0.137 | < 0.000001 |
| 2502.32 | 1.00 | 0.554 | 0.137 | < 0.000001 |
| 2257.10 | 0.39 | 0.46 | 0.158 | 0.000586 |
| 2212.17 | 34.05 | 0.032 | 0.214 | < 0.000001 |
| 1450.78 | 1.81 | 0.151 | 0.233 | < 0.000001 |
| 2286.15 | 0.84 | 0.443 | 0.295 | < 0.000001 |
| 1242.58 | 2.42 | 0.179 | 0.308 | < 0.000001 |
| 1538.78 | 1.29 | 0.377 | 0.361 | < 0.000001 |
| 1361.78 | 2.47 | 0.222 | 0.39 | < 0.000001 |
| 1029.65 | 3.45 | 0.466 | 0.821 | < 0.000001 |
| 1241.77 | 1.99 | 0.488 | 0.821 | < 0.000001 |
